# A High Sensitivity Preamplifier for Quartz Tuning Forks in QEPAS (Quartz Enhanced PhotoAcoustic Spectroscopy) Applications

**DOI:** 10.3390/s17112528

**Published:** 2017-11-03

**Authors:** Tomasz Starecki, Piotr Z. Wieczorek

**Affiliations:** Institute of Electronic Systems, Warsaw University of Technology, Nowowiejska 15/19, 00-665 Warsaw, Poland; tomasz@starecki.com

**Keywords:** quartz tuning fork, quartz enhanced photoacoustic spectroscopy, QEPAS sensor preamplifier design, transimpedance amplifier

## Abstract

All the preamplifiers dedicated for Quartz Enhanced PhotoAcoustic Spectroscopy (QEPAS) applications that have so far been reported in the literature have been based on operational amplifiers working in transimpedance configurations. Taking into consideration that QEPAS sensors are based on quartz tuning forks, and that quartz has a relatively high voltage constant and relatively low charge constant, it seems that a transimpedance amplifier is not an optimal solution. This paper describes the design of a quartz QEPAS sensor preamplifier, implemented with voltage amplifier configuration. Discussion of an electrical model of the circuit and preliminary measurements are presented. Both theoretical analysis and experiments show that use of the voltage configuration allows for a substantial increase of the output signal in comparison to the transimpedance circuit with the same tuning fork working in identical conditions. Assuming that the sensitivity of the QEPAS technique depends directly on the properties of the preamplifier, use of the voltage amplifier configuration should result in an increase of QEPAS sensitivity by one to two orders of magnitude.

## 1. Introduction

Trace gas sensors are used in numerous applications, such as environmental science (e.g., monitoring of atmospheric pollutants) [1], medical diagnostics (e.g., breath analysis) [2], industrial control [3] and the detection of dangerous substances (e.g., toxic gases or explosives) [4]. There are many methods that can be used to implement trace gas sensors. One of them, which is particularly well suited for such applications, is photoacoustic spectroscopy (PAS) [5,6,7].

In photoacoustic spectroscopy, the investigated gas is illuminated with light, and its wavelength is adjusted to the absorption line of interest. Absorption of light results in an increase of the local temperature and pressure. Modulation of the light intensity or wavelength with a given frequency *f* will thus lead to periodic temperature and pressure changes. Such pressure changes (i.e., photoacoustic signal) can be measured with a microphone or piezoelectric transducer. One of the most important facts about the induced photoacoustic signal is that its amplitude is proportional to the concentration of the absorbing gas. This allows the design of photoacoustic sensors that are capable of concentration measurements with the linearity of several orders of magnitude. For obvious reasons, solutions dedicated to trace gas detection applications are usually optimized towards the highest possible sensitivity, i.e., capability of detecting as low concentrations as possible. One of the methods of increasing sensitivity of the photoacoustic equipment is the use of an acoustic resonance for amplification of the photoacoustic signal. In such a case the signal gain is equal to the quality factor of the applied resonance [8]. A particular solution of that kind uses a quartz tuning fork (QTF) as the photoacoustic signal detector/transducer. For this reason the described modification of photoacoustic spectroscopy is called QEPAS (Quartz Enhanced PhotoAcoustic Spectroscopy) [8,9,10,11,12]. Due to the fact that QTFs may have quality factors of the order of 10^4^–10^5^ [8,13,14,15], a very high signal gain can be obtained. 

**Figure 1:**
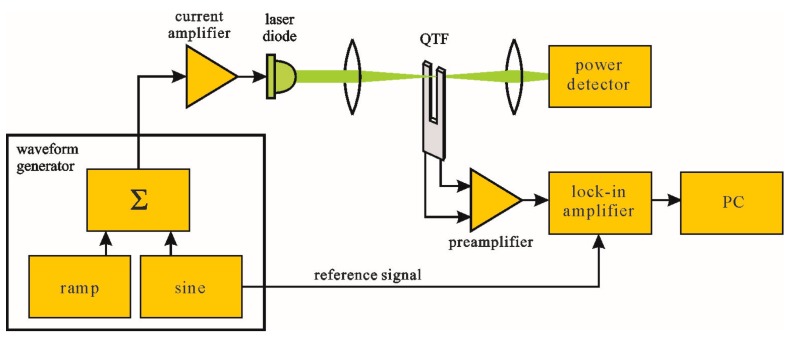
An example of a QEPAS setup block diagram.

A simplified block diagram of a QEPAS setup is presented in Figure 1. In the most common QEPAS implementation, a light beam produced with a laser diode is collimated between the QTF prongs. The laser diode is driven by a current amplifier, which in turn is controlled by a waveform generator. The output signal from the waveform generator consists of two components, slow ramp of relatively high amplitude and a small amplitude sine wave. Taking into consideration that wavelength of the light emitted by a laser diode depends on the current flowing through the diode, the slow ramp implements wavelength scanning, while the sine wave component results in additional small-deviation wavelength modulation. Absorption of the modulated light by the investigated gas produces local gas pressure changes, which periodically deflect prongs of the tuning fork synchronously to the light modulation. Each deflection of the prongs results in change of the charge/voltage at the resonator terminals (nodes). Such a signal is passed through a preamplifier, and then its amplitude is measured by a lock-in amplifier and can be recorded by a computer (PC). If the sine wave modulation frequency is appropriately selected, the QEPAS signal can be substantially amplified due to the resonance properties of the tuning fork.

All the preamplifiers dedicated to QEPAS applications that have been so far reported in the literature have been based on operational amplifiers working in transimpedance configurations [8,12,13,14,15,16]. However, QEPAS sensors are based on quartz tuning forks, and that quartz is a piezoelectric material with a relatively high voltage constant and relatively low charge constant [17]. This lead us to the conclusion that a transimpedance amplifier is probably not an optimal solution, and that properties of the preamplifier can be substantially improved if the preamplifier is implemented in a different way.

## 2. Preliminary Considerations

### 2.1. Preamplifiers Dedicated for Piezoelectric Sensors

Operation of the quartz tuning forks is based on a piezoelectric effect. A force applied to a piezoelectric material results in its mechanical deformation and produces an electric charge on certain opposite faces of the material [18,19]. Piezoelectric materials can be characterized by means of several properties, in particular by a piezoelectric charge constant and piezoelectric voltage constant. The first one reflects the electric polarization generated in a material per unit of mechanical stress applied to this material. The second, is defined as the electric field produced in a material per applied unit of mechanical stress. Values of the two constants and their ratio depend strongly on the material and may vary in a relatively wide range. To give an example: quartz has quite a high voltage constant (118 V·m/N) and a low charge constant (4.6 pC/N), while commonly used PZT (Lead Zirconate Titanate) has a lower voltage constant (38 V∙m/N), but its charge constant (580 pC/N) is over two orders of magnitude higher than the corresponding sensitivity of quartz [17]. 

**Figure 2:**
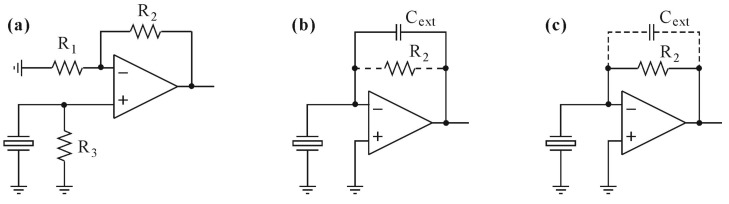
Basic configurations of preamplifiers dedicated for use with piezoelectric sensors: (**a**) voltage amplifier; (**b**) charge amplifier; (**c**) transimpedance amplifier.

Since a quartz tuning fork is a piezoelectric sensor, it should be considered as a high impedance signal source. Hence, the primary role of a preamplifier used in QEPAS equipment is conversion of the signal available at high-impedance terminals of the QTF to a low-impedance voltage signal source. Preamplifiers dedicated to piezoelectric sensors are usually implemented with operational amplifiers (op-amps) working as voltage amplifiers, charge amplifiers or transimpedance amplifiers (Figure 2) [20]. 

Resistor *R*_3_ used in the voltage amplifier circuit (Figure 2a) is required to supply bias current to the non-inverting input of the op-amp, and its value can be in the range of gigaohms if the input stage of the op-amp is built with Junction Field Effect Transistors (JFET) or Metal Oxide Semiconductor Field Effect Transistors (MOSFET). In such a case, the electrical load of the QTF is negligible and the sensor works in the voltage-emitting mode. Signal produced at the electrodes of the QTF is amplified with the *kf* gain of (1 + *R*_2_*/R*_1_). 

In the charge amplifier configuration (Figure 2b), a piezoelectric sensor is connected to the inverting input of the operational amplifier. Due to the feedback loop of the op-amp formed by the *C_ext_* and *R*_2_ that are connected in parallel, voltage at the inverting input is very close to the ground (zero voltage). This means that voltage across the sensor electrodes is forced to be virtually zero, and the sensor works in the charge-emitting mode. Obviously, the charge changes induced by the sensor can be considered as a current flowing through the parallel connection of *C_ext_* and *R*_2_, producing voltage signal at the output of the amplifier [19,21,22]. Resistor *R*_2_ is used to ensure the inverting input polarity for DC voltage, limit the voltage gain and prevent from output voltage saturation (as input offset voltage appears at the output amplified with the voltage gain). The *R*_2_ resistor also influences the bandwidth of the amplifier, by setting the upper bandwidth frequency to the value of (1/2π·*R*_2_*·C_ext_*). The charge amplifiers are used mainly with the sensors of relatively high inherent capacitance *C_s_*, because voltage gain *kf* of the charge amplifier is defined by the *C_s_/C_ext_* ratio. Taking into consideration that the equivalent capacitance of a quartz tuning fork is of the order of a few picofarads, and that the stray (parasitic) capacitance of the circuit is of a similar level, obtaining high gains of the QTF signal working in the charge amplifier configuration is not possible. The main reason for using a capacitor in the feedback loop of charge amplifiers is that if the sensor is a capacitive signal source, its impedance will change with the frequency. Thus, the use of an ordinary inverting amplifier with a resistor in the feedback loop will result in a frequency dependence of the gain. If both source and feedback impedances are capacitors, the gain will not vary with the frequency. However, if the signal source produces a sine wave of a fixed frequency *f*, the capacitor in the feedback loop can be replaced by a resistor *R*_2_ of appropriately selected value (*R*_2_ = *kf*/2π·*f*·*C_s_*), converting a charge amplifier into a transimpedance amplifier (Figure 2c). It can be easily noticed from the above description, that the structure of the transimpedance amplifier is identical to the charge amplifier (Figure 2b). The only difference is that the main feedback loop component that sets the gain of such a circuit is *R*_2_, while *C_ext_* is a stray capacitance (or is a capacitor used to limit the signal bandwidth). Obviously, a transimpedance amplifier applied as a QTF signal amplifier has similar drawbacks as the charge amplifier.

As already mentioned, it seems as though all the QTF preamplifiers used in QEPAS applications described in the literature have been so far implemented as transimpedance amplifiers [8,9,10,11,12,16]. Moreover, the cited solutions used feedback resistors *R*_2_ of relatively high values; the most common of which was 10 MΩ [8,10,16].This was probably done to obtain a high gain factor; however, if we assume values of *R*_2_ = 10 MΩ and stray capacitance *C_ext_* = 2.5 pF, we obtain a resulting upper limit frequency of approx. 6.4 kHz. This in turn means that virtually all the cited QEPAS preamplifiers that were used with the 32.768 kHz QTFs were working outside the preamplifier bandwidth, and thus the QEPAS signal was subjected to substantial attenuation.

### 2.2. Quartz Tuning Fork Model 

**Figure 3:**
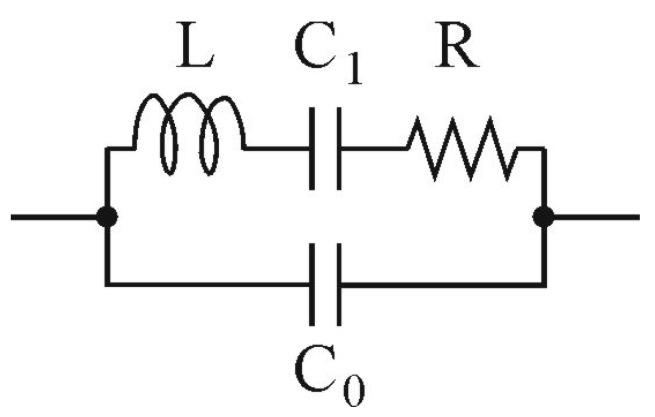
Butterworth–Van Dyke model of a quartz crystal resonator.

QEPAS sensors are based on quartz tuning forks working in resonance conditions. Hence, theoretical analysis of a QEPAS preamplifier requires use of a QTF model that would reflect the resonance properties of the fork. This can be obtained with a Butterworth–Van Dyke model (Figure 3) [13,14,15]. 

In the above figure, the resistor *R* models the energy losses, the capacitor *C*_1_ and the inductor, *L*_1_ represent the potential and the kinetic energy storage, and the parallel capacitor *C*_0_ corresponds to the total stray capacitance of the electrodes, crystal enclosure, leads, etc. Obviously, in order to model behavior of a preamplifier used with a given QTF, all four component values (*C*_0_, *C*_1_, *L*_1_ and *R*) for this particular resonator must be estimated first. Probably the most straightforward way to extract the parameters is to measure the admittance of the QTF vs. frequency, and perform some calculations, taking into consideration that [13,14]:

1. series resonant frequency *fs* can be easily , measurements as a frequency at which the QTF has the highest admittance;
2. series anti-resonant frequency *fa* can be easily determined from the measurements, as at this frequency the QTF has the lowest admittance;
3. *C*_0_ for the quartz crystals is usually of a few pF and its typical value for the given resonator is specified by the manufacturer in the datasheet. For 32.768 kHz crystals we can assume *C*_0_ = 2.5 pF;
4. knowing that:
  (1) $$fs=\frac{1}{2\pi \sqrt{L_{1}\cdot C_{1}}}$$
  and:
  (2) $$fa=1/2\pi \sqrt{L_{1}\frac{C_{0}\cdot C_{1}}{C_{0}+C_{1}}}$$
  we get *C*_1_ as:
  (3) $$C_{1}=C_{0}\cdot \left({\left(\frac{fa}{fs}\right)}^{2}-1\right);$$
  and then *L* can be calculated from (1);
5. quality factor (*Q*) of the crystal can be estimated from the measured crystal admittance vs. frequency values, taking into consideration that:
  (4) $$Q=\frac{fs}{\Delta f_{-3dB}}$$
6. Finally we can obtain *R* from:
  (5) $$R=\frac{2\pi fs\cdot L_{1}}{Q}.$$

### 2.3. Quartz Tuning Fork Measurements 

**Figure 4:**
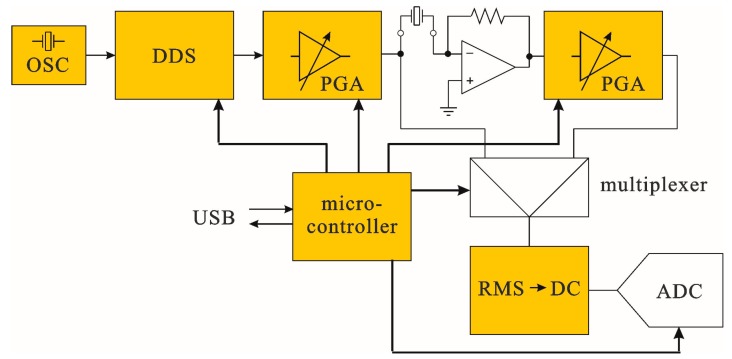
Block diagram of a system used for QTF characterization.

As described above, estimation of the component values used in the QTF model requires determination of the QTF admittance vs. frequency characteristics. In order to evaluate such properties of the QTF used in our experiments, we designed a dedicated system, whose block diagram is given in Figure 4.

**Figure 5:**
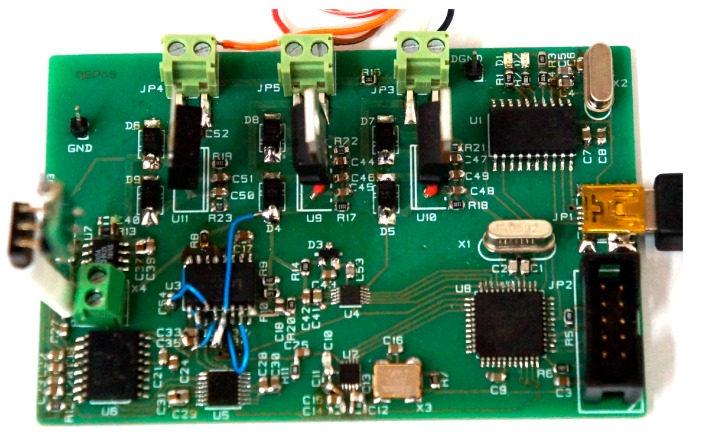
A photo of the designed system during QTF measurements.

**Figure 6:**
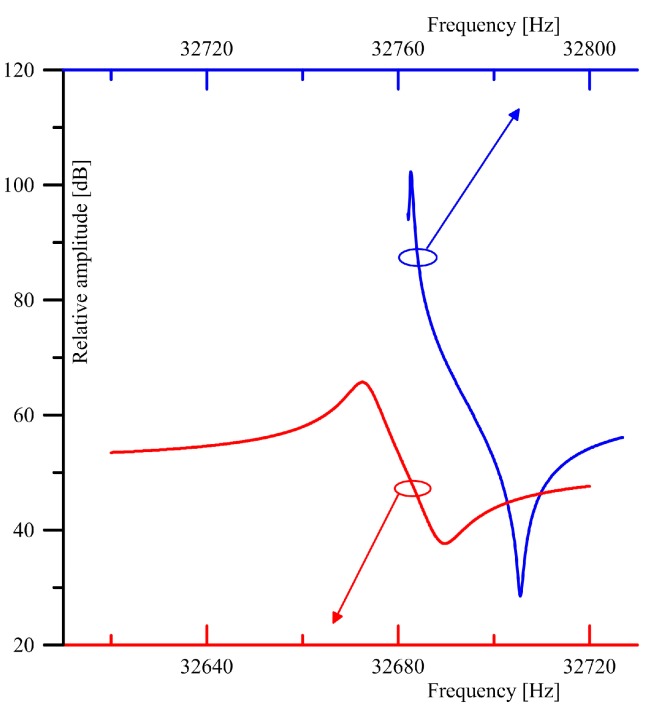
QTF admittance vs. frequency measured with a frequency step of 0.1 Hz before (upper plot) and after (bottom plot) of the QTF package cup removal (32.768 kHz crystal resonator, model LFXTAL002996Bulk, manufactured by IQD Frequency Products).

The system was controlled by a PC computer (via USB interface). As a result, substantial parts of the control software, numerical calculations and user interface were implemented on the PC, and thus it was possible to have the hardware and firmware of the system significantly simplified. Admittance of the crystal was measured point-by-point for a given (programmable) range of frequencies. As can be seen from the Figure 4, signal from a programmable sine generator, implemented with a DDS (Direct Digital Synthesis) circuit, was applied to a transimpedance amplifier, in which the output amplitude is proportional to the admittance of the crystal for a given frequency. The DDS circuit with a reference clock based on a crystal oscillator (OSC) produces a high quality sine wave with a precisely known (and programmable) amplitude and frequency. Due to expected very high quality factors of the QTFs, resulting in very narrow resonance curves, we used a DDS circuit with a frequency resolution of the output sine wave better than 0.1 Hz. In order to obtain high dynamic range of the measurements, the signal from the DDS generator is passed through a low-noise, high-precision PGA (Programmable Gain Amplifier) whose gain is digitally programmable in the range of −95.5–+31.5 dB with a 0.5 dB resolution. Signal from the output of the transimpedance amplifier is passed through an identical PGA. Amplitude of the stimulating and output signals is measured with a true RMS (root mean square) converter. Switching between the two signals is implemented with a multiplexer. A DC voltage at the output of the true RMS converter is proportional to the RMS value of the input signal, and is measured with a high-resolution ADC (analog-to-digital converter). All the settings of both PGA channels, DDS generator, multiplexer and ADC are controlled by a microcontroller, which acts according to the commands sent via USB from the PC computer. A photo of the system performing measurements is given in Figure 5. Admittance vs. frequency dependence of the QTF used in further experiments, and measured with the presented system is given in Figure 6. The measurements clearly show that removal of the crystal package cup (necessary for QEPAS experiments) results in a slight decrease in the resonance frequency and substantial drop of the quality factor of the resonator.

## 3. Theoretical Analysis of the Preamplifier Noise Properties

The system described in the previous section was used to determine the *fs* and *fa* frequencies of the QTF, operating under atmospheric pressure conditions in ambient temperature. This, in turn allowed us to determine the *L*_1_ and *C*_1_ parameters for known *C*_0_, giving *C*_1_ = 5.95 fF and *L*_1_ = 4768 H, which seem to be within the typical range according to [13,14]. Moreover, due to the sufficient frequency resolution of the system, we also estimated *Q* factor according to (4), and as a result we were able to estimate the value of *R* = 190 kΩ. 

**Figure 7:**
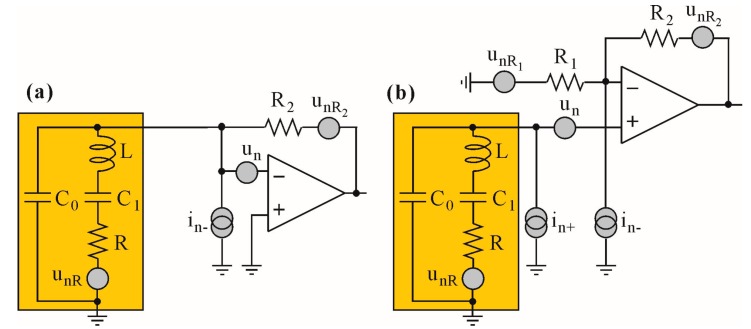
Noise sources of the analyzed configurations of the preamplifier: (**a**) transimpedance configuration; (**b**) voltage configuration.

Since in QEPAS applications the QTF operates in close *fs* proximity, it acts as a device in which the reactances of *L*_1_, *C*_1_ and *C*_0_ do not contribute to the input parameters of preamplifier circuits shown in Figure 7. Such parameters as gain, noise or sensitivity depend solely on *R*, *R*_1_, *R*_2_ and noise sources.

The electrical macromodels shown in Figure 7 consist of voltage and current noise sources, i.e., *u_n_*, *i_n+_*, *i_n−_* (representing amplifier noise sources), an ideal operational amplifier, thermal noise sources of the resistors *R*_1_ and *R*_2_ (*u_nR_*_1_ and *u_nR_*_2_, respectively) and the QTF model (see Figure 3). The QTF model was enhanced with a noise source *u_nR_*, which represents both the thermal and flicker noise of the QTF [23].

**Figure 8:**
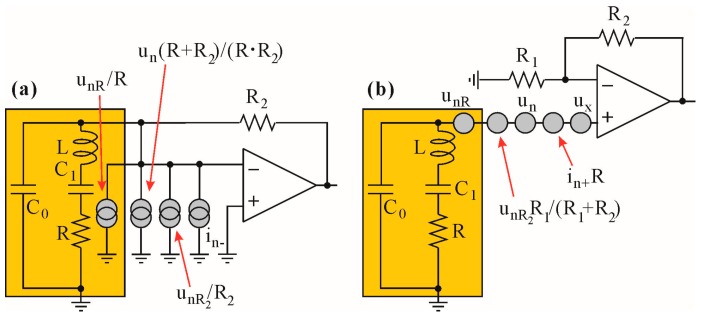
Circuits presented in Figure 7 with the noise sources transformed to the amplifier inputs: (**a**) transimpedance configuration; (**b**) voltage configuration.

In order to estimate and compare the overall contribution of noise sources in macromodels of transimpedance and voltage topologies from Figure 7, we have transformed all sources to either current (Figure 8a) or voltage sources (Figure 8b) referred to the amplifier inputs. It is worth mentioning that the noise model of the charge amplifier is identical to the transimpedance one with respect to circuit 20’s gain and bandwidth, which mainly result from the *C_ext_* connected in parallel to *R*_2_ (see Figure 2b), where *kf* = (*C*_0_ + *C*_1_)/*C_ext_*.

In order to transform the sources, additional arithmetic operations were performed. The *u_nR_*_2_ source was transformed from the amplifier’s output to its inverting input (see Figure 8a). For this reason its amplitude was divided by the close loop transimpedance gain (*ki*) of the amplifier. The voltage noise *u_n_* source is placed between the feedback node and the inverting input. However, from a mathematical point of view, it can be treated as a voltage source connected to a non-inverting input. Therefore, its contribution to the feedback node results from voltage gain of the close loop amplifier, divided by transimpedance gain, i.e., (*R*_2_ + *R*)/*R*_2_*·R*. This way, the *u_n_* source is transformed to the feedback node and converts into a noise current source connected in parallel to the amplifier’s noise *i_n−_* source. Therefore, assuming the independence of noise processes, the total current variance *i_tot_* referred to the input node (QTF) can be derived as:

(6) $$i_{tot}{}^{2}=i_{n-}{}^{2}+\frac{1}{R^{2}}\cdot \left(u_{nR}{}^{2}+u_{n}{}^{2}\right)+\frac{1}{R_{2}{}^{2}}\cdot \left(u_{nR2}{}^{2}+u_{n}{}^{2}\right)$$

The overall variance of voltage noise sources referring to the non-inverting input of the circuit in Figure 8b can be derived when all of the noise sources are converted to the voltage sources referred to the input. Therefore, we have converted the *i_n-_* and *u_nR_*_1_ sources to current sources connected in parallel to *R*_1_, and furthermore, we transferred these sources to the non-inverting input as voltage source *u_x_*, i.e.,:

(7) $$u_{x}=\left(\frac{u_{nR1}}{R_{1}}+i_{n-}\right)\cdot \frac{R_{1}\cdot R_{2}}{R_{2}+R_{1}}$$

The remaining *u_nr_*_2_ source can be easily transferred to the non-inverting input by dividing its amplitude by the voltage close loop gain *kf* of the circuit in Figure 7b, i.e., *kf* = (1 + *R*_2_*/R*_1_). This way we obtained a compact form of variance of the input voltage source of the circuit in Figure 8b, as derived in (8):

(8) $$u_{tot}{}^{2}=u_{nR}{}^{2}+{\left(i_{n+}R\right)}^{2}+u_{n}{}^{2}+\frac{u_{nR2}{}^{2}\cdot R_{1}{}^{2}+u_{nR1}{}^{2}\cdot R_{2}{}^{2}+i_{n-}{}^{2}{\left(R_{2}\cdot R_{1}\right)}^{2}}{{\left(R_{1}+R_{2}\right)}^{2}}$$

The qualitative comparison of total noise described in (6) and (8) requires some simplifications. First of all, the output noise of both architectures shown in Figure 8 results from amplification of (6) and (8) by the transimpedance (*ki* = *R*_2_) and voltage (*kf*) gain, respectively. Moreover, some of the factors in (6) and (8) can be neglected due to the major influence of other factors. Therefore, (6) multiplied by *ki* can be estimated by (9), whereas (8) leads to (10) when multiplied by *kf*:

(9) $$u_{nouttrans}{}^{2}\approx \frac{R_{2}{}^{2}}{R^{2}}\cdot \left(u_{nR}{}^{2}+u_{n}{}^{2}\right)+i_{n-}{}^{2}\cdot R_{2}{}^{2}$$

(10) $$u_{noutvolt}{}^{2}\approx \frac{{\left(R_{2}+R_{1}\right)}^{2}}{R_{1}{}^{2}}\cdot \left(u_{{}_{nR}}^{2}+u_{n}^{2}\right)+i_{n+}^{2}\cdot {\left(\frac{R\;(R_{1}+R_{2})}{R_{1}}\right)}^{2}+i_{n-}^{2}\cdot R_{2}^{2}+u_{nR1}^{2}\cdot {\left(\frac{R_{2}}{R_{1}}\right)}^{2}$$

One can see that the output noise derived in (10) contains two additional components, i.e.,:

(11) $$i_{n+}{}^{2}\cdot {\left(\frac{R\;(R_{1}+R_{2})}{R_{1}}\right)}^{2}+u_{nR1}{}^{2}\cdot {\left(\frac{R_{2}}{R_{1}}\right)}^{2},$$

which result from the presence of an additional resistor *R*_1_ and the obvious fact that the non-inverting input is not connected to the ground. However, the total noise in both cases (i.e., transimpedance and voltage topology) is strictly determined by either the values of *R*_2_ and *R*_2_*/R* (so obviously *ki* and the *ki* to series resonance resistance ratio values) or the *R*_2_*/R*_1_ values (where *R*_2_*/R*_1_ ≅ *kf*). Therefore, for (*R*_2_*/R*) ≈ (*R*_2_*/R*_1_) and *R*_2_ much greater than *R*_1_, the overall noise is slightly higher in the case of the voltage op-amp topology, due to the presence of additional *i_n__−_* and *u_nR_*_1_ sources. However, it is a rather small difference.

## 4. Measurements 

**Figure 9:**
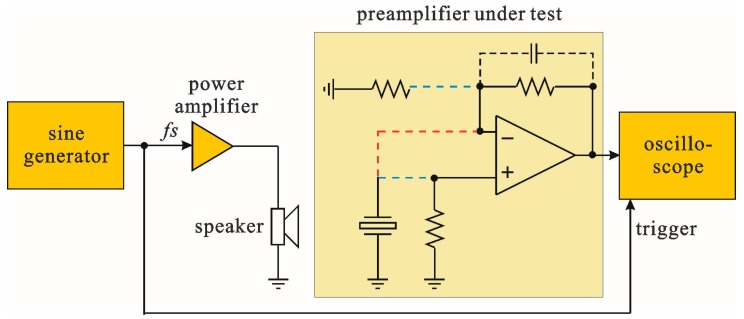
Block diagram of a system used for experimental verification of the preamplifier configurations (voltage, charge and transimpedance).

In order to experimentally evaluate properties of the preamplifier configurations discussed in this paper, it was necessary to measure and compare output signals from preamplifiers working with identical mechanical stimulation of the tuning fork. The measurements were performed in a circuit whose block diagram is presented in Figure 9. The tuning fork was stimulated by means of pressure changes produced by a high-frequency speaker. The preamplifier was implemented in such a way that changes between the voltage, charge or transimpedance configurations were possible by swapping three connections made with short, flexible cables (marked with dashed lines). As a result, all preamplifier configurations were based on the same operational amplifier and the same tuning fork. Moreover, positions of the speaker and the tuning fork were fixed, which resulted in exactly identical conditions during measurements of preamplifiers. Signals from the preamplifiers’ outputs were observed and measured with a Tektronix MDO3024 oscilloscope. 

## 5. Measurements of QTF Source Parameters

The investigated circuits shown in Figure 2 and Figure 9 were implemented in the Texas Instruments Analog System Lab Kit PRO. We stimulated the QTF connected to the input of voltage amplifier, charge amplifier and transimpedance amplifier with identical amplitude of the acoustic signal (as described in previous subsection) at the *fs* frequency. This way, we were able to estimate the amplitude of the voltage and current signals (in the case of the voltage amplifier and charge/transimpedance topology, respectively) for a particular acoustic signal level. Since the voltage and charge/transimpedance amplifier topology represent opposite conditions, i.e., theoretically infinite input impedance (for voltage amplifier) and zero impedance (for charge and transimpedance amplifier) of the QTF load, we were able to estimate the Thevenin model’s parameter of the QTF at the *fs* frequency. 

Any capacitance *C_ext_* connected in parallel with *R*_2_ reduces the bandwidth of the amplifier in both topologies. Therefore, it is not recommended to use *R*_2_ of the order of several megaohms, because in such a case the upper limit frequency:

(12) $$f_{u}=\frac{1}{2\cdot \pi R_{2}C_{ext}}$$

is solely defined not only by the bandwidth of the op-amp itself, but by the parasitic capacitances present in the circuit. The use of moderate voltage and transimpedance gain (e.g., *kf* = 21 V/V and *ki* = 100 kΩ respectively) eliminates the influence of the op-amp parameters on the waveforms at the *fs* frequency. Therefore, in the experiment *R*_1_ = 4.7 kMΩ and *R*_2_ = 100 kMΩ were assumed. In the case of the charge amplifier, the gain is defined by the (*C*_0_ + *C*_1_)/*C_ext_* ratio. One can see that it is difficult to obtain high gain ratios in the case of the charge amplifier due to extremely low *C_ext_* values. In the case of our QTF, in order to obtain gain close to 6 V/V, it was necessary to use *C_ext_* = 400 fF, whereas parasitic capacitances of this order are present on the board. Therefore, extreme care must be taken during the design of the charge amplifier PCB. The resistor *R*_2_, which is only necessary for bias in the charge configuration, was 100 MΩ.

**Figure 10:**
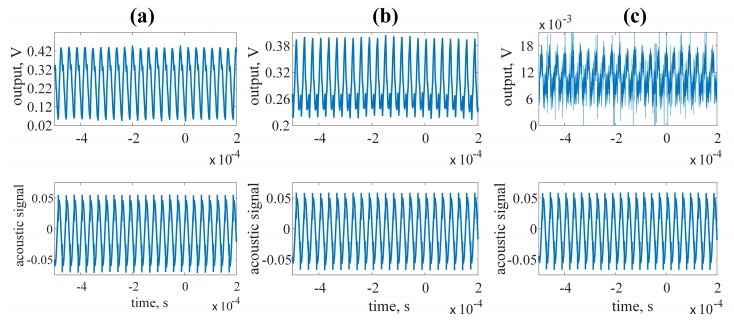
Output signals observed at the output the investigated preamplifier working in different configurations: (**a**) voltage amplifier; (**b**) charge amplifier; (**c**) transimpedance amplifier.

Figure 10a–c show the output waveforms of the op-amp working in the voltage, charge and transimpedance amplifier configurations respectively. The acoustic signal was obtained from a high frequency speaker connected to a power amplifier. The output power was 0.7 W, the operating frequency was set to *fs* and the speaker was placed in close proximity of the QTF (4 cm). The obtained AC amplitudes were *u_v_* = 208 mV and *u_i_* = 6.5 mV for the voltage and transimpedance amplifier respectively. In the case of the charge amplifier configuration the output amplitude was 90 mV.

**Figure 11:**
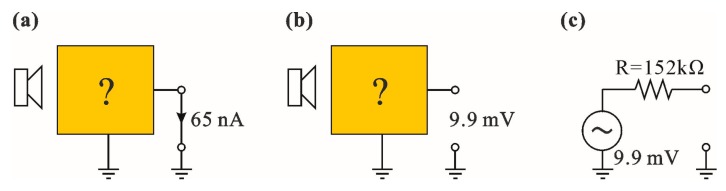
Black box method of signal source model evaluation: (**a**) current measurement at short circuit signal source; (**b**) voltage measurement at open circuit signal source; (**c**) resulting signal source model with the amplitude of 9.9 mV and internal resistance of 152 kΩ.

At resonance the influence of reactances on the QTF impedance is negligible. For this reason, the QTF can be represented as an ordinary non-ideal voltage or current source, depending on the topology of the amplifier (in this case the voltage or transimpedance respectively). Knowing the amplitudes of output waveforms (Figure 10), we were able to estimate the short circuit current *ui*/*ki* = 65 nA (Figure 11a), and the open circuit amplitude *u_v_*/*kf* = 9.9 mV (Figure 11b). This way, we estimated the internal resistance *R* of the Thevenin source representing QTF (Figure 11c), i.e., *R* = (*u_v_*·*ki*)/(*ku*·*ui*) = 152 kΩ. One can see that the measured *R* value is close to the estimate based on (4) and (5), i.e., 190 kΩ.

The measurements clearly showed that voltage and transimpedance topologies enforce different behavior of the QTF source. The open circuit voltage *u_v_*/*kf* = 9.9 mV is easy to detect and amplify with an ordinary op-amp, such as TL08x or TL07x used in the experiment. However, in the short circuit mode of the QTF operation (transimpedance topology), one needs at least *ki* = *R*_2_ = 152 kΩ of the transimpedance gain in order to obtain the same signal level (9.9 mV) as the one present at the open circuit QTF nodes (without any amplifier at all).

The measurements performed with QTF stimulated by the acoustic signal are valuable, since in the QEPAS technique, QTF is used exactly in the same way, as an acoustic to electrical signal transducer. Let us compare the empirical behavior of QTF in both considered preamplifier topologies, i.e., voltage and transimpedance. In the voltage topology, we obtained *u_v_* = 208 mV for *kf* = 21 V/V (*R*_1_ = 4.7 kΩ and *R*_2_ = 100 kΩ). In order to obtain the same output signal in the case of the transimpedance topology, we should apply *R*_2_ = 208 mV/65 nA = 3.2 MΩ. From now on, the (9) and (10) seem to be more obvious options. The input current noise *i_n−_* has to be amplified over an order of magnitude in the case of the transimpedance topology (i.e., *R*_2_ = 3.2 MΩ for transimpedance and *R*_2_ = 100 kΩ for the voltage mode topology for the identical output *u_v_*). Therefore, even though the *i_n+_* in (10) is present, its contribution is negligible due to significantly lower *R*_2_ in the case of the voltage topology. Moreover, the ((*u_n_*_R_^2^ + *u_n_*^2^)·*R*_2_^2^/*R*^2^) term is much higher in the case of transimpedance configuration. First of all, *u_n_*_R_^2^ results from the thermal noise, which is proportional to the value of *R*, however *R*_2_ is 32 times higher in comparison to the voltage topology. Moreover, in order to obtain the same level of signal at the outputs of both topologies, (*ki*/*R*) ≅ (((*R*_2_ + *R*_1_)/*R*_1_)), which obviously enhances influence of the thermal *R*_2_ noise. One can see that if the presented case study is applied to both topologies, the noise level is approximately an order of magnitude higher for the same output signal level in the case of the transimpedance topology. For this reason the rough signal-to-noise ratio (*SNR*) is an order of magnitude higher for the voltage preamplifier topology. 

In order to confirm the theoretical considerations we performed two types of measurements for all three topologies. In the first one, with the MDO3024 oscilloscope, we recorded m = 10^7^ samples for each topology with and without the acoustic signal applied. The bandwidth of the measurements was limited to 500 kHz, and AC coupling was used. In the second case, we utilized the MDO3024 with additional spectrum analyzer. In this case, the bandwidth was only 10 kHz–100 kHz, whereas the resolution bandwidth (RBW) was 50 Hz. 

To calculate the signal-to-noise ratio (*SNR*) in the first case, we used Equation (13), where *u_i_* stands for subsequent signal samples from the output of the preamplifier, and *un_i_* stands for the samples recorded without the acoustic stimulation:

(13) $$SNR\;=\;20\log_{10}\frac{\sqrt{\frac{1}{m}\sum_{i=1}^{m}u_{i}{}^{2}}}{\sqrt{\frac{1}{m}\sum_{i=1}^{m}{un}_{i}{}^{2}}},\;m\;=\;10^{7}$$

**Figure 12:**
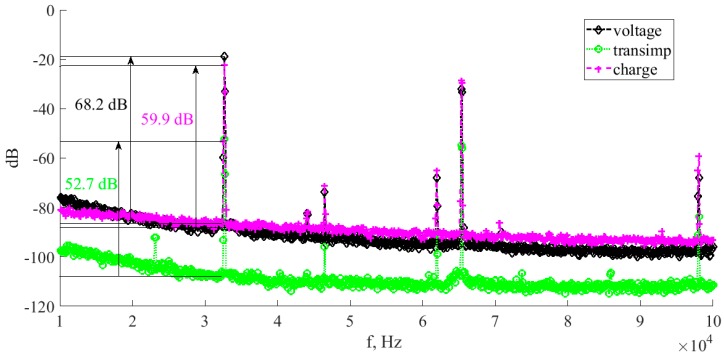
Average spectra obtained for the voltage, charge and transimpedance topologies. The *SNR* values in the neighborhood of the *fs* frequency are marked.

Our second proposed technique for an *SNR* assessment was to estimate the signal to noise ratio in the narrow bandwidth neighborhood of the *fs* frequency. For this purpose, we registered output power spectra of the voltage, transimpedance and charge amplifiers. The results are shown in Figure 12 and Table 1.

Results of both mentioned methods used to estimate wideband and narrowband *SNR* of all three analyzed preamplifier topologies are given in Table 1.

The signal-to-noise values in Table 1 prove previous considerations regarding voltage and transimpedance topologies. One can see that the *SNR* in the case of voltage preamplifier is 18.5 dB higher in comparison to the transimpedance configuration.

Narrow bandwidth *SNR* estimates show better results for all considered topologies. In such a case, EMI influence is minimized to a very narrow part of the spectrum, which is similar to the 1/f noise. However, it turns out that in the case of the voltage preamplifier, the *SNR* yields 15.5 dB improvement over the transimpedance topology (*SNR* = 68.2 dB, 59.9 dB and 52.7 dB for the voltage, charge and transimpedance topologies, respectively).

## 6. The Proposal of the High Sensitivity Preamp–Discussion

The theoretical consideration and measurement results led us to the concept of a multistage high sensitivity preamp dedicated for the QEPAS. First of all, the voltage amplifier topology seems to be the obvious choice when the noise and signal amplitudes are considered. Moreover, due to rather high *R* values, the input stage of the amplifier should be fully differential, so that any external electromagnetic (EM) disturbances would not interfere with the weak signal available at the QTF nodes. The input resistance of the op-amp should be of at least an order of magnitude higher than QTF’s *R*. Since the *R* value is high, we suggest guard shielding, which minimizes leakage of the input voltage stage. In the tested solution, we used AD 623 instrumentation amplifier at the first stage.

**Figure 13:**
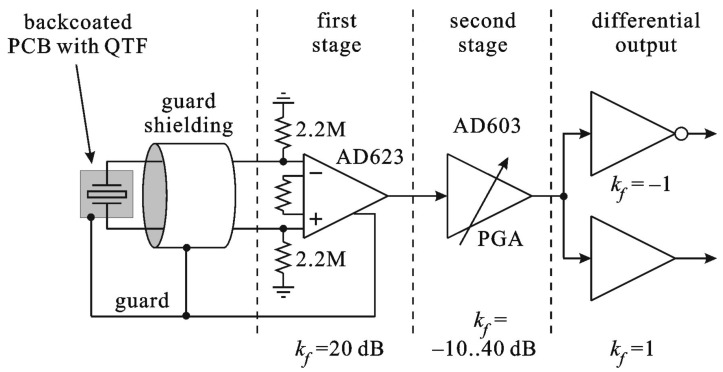
Block diagram of the proposed preamplifier.

The scenario of the acoustic stimulation used in our measurements was rather optimistic, since we used a powerful source of acoustic signal. In real QEPAS applications, the required *kf* gain of the op-amp is unknown, due to unknown concentration of the measured gas solution. Therefore, the preamplifier should have a variable gain stage. Figure 13 shows a block diagram of the proposed QEPAS multistage preamplifier.

**Figure 14:**
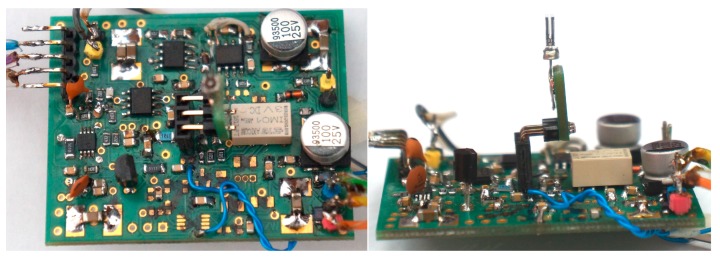
Photos of the assembled preamplifier with QTF used for measurements.

In order to minimize the noise contribution to the system presented in Figure 13, we used a PGA (AD 603 dedicated for radio frequency applications) with externally limited bandwidth as a middle stage. This way the noise amplitude added to the signal of the first stage is minimized. The third stage of the designed preamplifier contains two amplifiers, i.e., inverting and non-inverting, in order to produce differential output signal. This way, the output signal is insensitive to additive electromagnetic interferences (EMI). The printed circuit board (PCB) of the proposed solution is shown in Figure 14. 

The proposed amplifier circuit was assembled on a 50 mm × 38 mm × 1.55 mm two-layer PCB made from an FR-4 material. Whereas the bottom layer of the PCB consists mainly of a ground shielding and some supply nodes, the top layer is used for signal paths. The AD623 and AD603 circuits are located in the top-middle part of the PCB (see Figure 14), in close proximity to the QTF connections. The differential amplifier output stage is located at the left edge of the board. This way, we minimized the asymmetric signal path, and therefore, reduced EMI influence. The circuit was subjected to the *SNR* measurements as described previously. The first method utilizing (13) yielded *SNR* = 37.13 dB, and the second one (i.e., with the narrow band spectrum analysis) gave *SNR* = 64 dB. Narrowband *SNR* is slightly worse in comparison to the presented measurements of the basic preamplifier structures due to the fact that the preamplifier was designed as a high-dynamic and programmable circuit that could be used in practical QEPAS applications. Such an approach resulted in a much more complex solution, with additional components that slightly increased noise level. Better wideband *SNR* results were obtained from the use of differential signal path, which reduces influence of EMI. 

## 7. Conclusions

In this paper, we presented both theoretical and experimental comparisons of the transimpedance preamplifier (so far the only solution used in QEPAS circuits), charge preamplifier and voltage preamplifier. The theoretical analysis, as well as the preliminary measurements, proved that the proposed voltage preamplifier solution results in a much higher output signal and better signal-to-noise ratio. The improvement over transimpedance circuits is at the level of one order of magnitude. Hence, the use of the voltage amplifier configuration should substantially increase sensitivity of the QEPAS technique.

## Figures and Tables

**Table 1:** Signal-to-noise ratio (*SNR*) for all considered topologies measured with 500 kHz and narrow bandwidth.

	Wideband *SNR* (dB)	Narrowband *SNR* (dB)
Voltage amplifier	29.74	68.2
Charge amplifier	23.29	59.9
Transimpedance amplifier	11.21	52.7
